# Incidence, Predictors, and Clinical Outcomes of Postoperative Cardiac Tamponade in Patients Undergoing Heart Valve Surgery

**DOI:** 10.1371/journal.pone.0165754

**Published:** 2016-11-17

**Authors:** Seng Chan You, Chi Young Shim, Geu-Ru Hong, Darae Kim, In Jeong Cho, Sak Lee, Hyuck-Jae Chang, Jong-Won Ha, Byung-Chul Chang, Namsik Chung

**Affiliations:** 1 Cardiology Division, Severance Cardiovascular Hospital, Yonsei University College of Medicine, Seoul, Republic of Korea; 2 Division of Cardiovascular Surgery, Severance Cardiovascular Hospital, Yonsei University College of Medicine, Seoul, Republic of Korea; Universita degli Studi di Roma La Sapienza, ITALY

## Abstract

This study aimed to investigate the incidence, predictors, and clinical outcomes of cardiac tamponade after heart valve surgery. A total of 556 patients who underwent heart valve surgery in a single tertiary center between January 2010 and March 2012 were studied. All patients underwent transthoracic echocardiography (TTE) about 5 days after surgery and TTE was repeated regularly. Patients with suspected acute pericardial hemorrhage were excluded. Cardiac tamponade occurred in twenty-four (4.3%) patients and all underwent surgical or percutaneous pericardial drainage. The median time of pericardial drainage after surgery was 17 (interquartile range, IQR, 13–30) days. Infective endocarditis, mechanical valve replacement of aortic or mitral valve, and any amount of pericardial effusion (PE) on the first postoperative TTE were related to the occurrence of cardiac tamponade (all p<0.05). After multivariate adjustment, occurrence of cardiac tamponade was associated with any amount of PE on the first postoperative TTE (hazard ratio, HR, 14.00, p<0.001) and mechanical valve replacement (HR 2.69, p = 0.025). The mean hospital days in patients with cardiac tamponade was higher than those without (34.9 vs. 13.5, p = 0.031). After pericardial drainage, there was no echocardiographic recurrence of significant PE during a median of 34.8 (IQR 14.9–43.7) months after surgery. Cardiac tamponade after heart valve surgery is not uncommon. Patients with any amount of PE at the first postoperative TTE or mechanical valve replacement should receive higher attention with regard to the occurrence of cardiac tamponade. Although it prolongs hospital stay, cardiac tamponade exhibits a benign clinical course without recurrence after timely intervention.

## Introduction

Pericardial effusion (PE) after cardiac surgery is a commonly encountered finding, even in patients without evidence of postoperative pericardial hemorrhage. It is known to be related to an inflammatory process of the pericardium, called post-pericardiotomy syndrome (PPS) [1]. Although it mostly exhibits a benign course without clinically serious complications, it is able to progress to potentially lethal cardiac tamponade which is associated with increased morbidity and mortality [2–5]. A few studies demonstrated that postoperative PE or consequent constrictive physiology were prevalent in patients undergoing coronary artery bypass grafting (CABG) [6,7]. However, there would be considerable variations in the incidence of PE and the consequent clinical course according to the types of surgery, underlying diseases, or the use of anticoagulants in patients undergoing heart valve surgery. Therefore, we sought to investigate the incidence and clinical courses of PE and cardiac tamponade. Moreover, we tried to define clinical or echocardiographic predictors of cardiac tamponade occurrence following heart valve surgery.

## Materials and Methods

The study consisted of a retrospective analysis of 556 consecutive adult patients who underwent open heart surgery because of valvular heart disease from January 2010 through March 2012 at a single tertiary referral center. Medical records as well as serial echocardiographic data following valvular heart surgery were reviewed. For all patients, the etiologies of valvular heart disease, severities of valvular dysfunction, types of surgery (repair or replacement), and concomitant surgery such as aorta surgery, CABG, or MAZE operation for atrial fibrillation were reviewed. All the patients in this study underwent mandatory anticoagulation as recommended by the guidelines, except for those with serious bleeding complications [8]. The length of stay in hospital was calculated in each patient from valvular operation to discharge. The patients were divided into two groups according to the occurrence of cardiac tamponade in the post-operative period. Those patients with cardiac tamponade were further classified into two subgroups based on the timing of its occurrence: early cardiac tamponade versus delayed cardiac tamponade. Early cardiac tamponade was defined as cardiac tamponade occurring earlier than 30 days after surgery. In contrast, delayed cardiac tamponade was defined as that occurring 30 days or later after surgery.

The laboratory findings including cardiac biomarkers, inflammatory markers, and coagulation profiles were reviewed in every patient. The peak values of post-operative white blood cell count (WBC), creatine kinase (CK), CK-MB isoenzyme, C-reactive protein (CRP), activated partial thromboplastin time (aPTT), and prothrombine time (PT) were analyzed. This study was approved by the Institutional Review Board of Yonsei University, Severance Hospital, Seoul, Korea. Written informed consent was exempt by the board as this study was retrospective in design. Patient records/information was anonymized and de-identified prior to analysis.

All subjects underwent comprehensive transthoracic echocardiography (TTE) using commercially available equipment prior to valvular heart surgery to assess the etiology of valvular disease, severity of valvular dysfunction, and cardiac function. Standard two-dimensional and Doppler measurements were performed per the recommendations of the American Society of Echocardiography guidelines [9]. The first follow-up TTE after heart valve surgery was performed on all patients at median 5 days (interquartile range, IQR, 4–7) after the surgery. Subsequently, follow-up TTE was performed regularly or based on clinical decision. In general, if the patients were uneventful, the second follow-up echocardiography was performed at 6 months after the surgery, and then annually for the evaluation of postoperative cardiac remodeling and serial follow-up of the valve function. However, if the patients exhibited uncertain signs or symptoms, more frequent TTE follow-up was performed based on the clinician’s decision. PE was classified into three grades as follows: small, 10–14mm of loculated effusion or <10 mm of circumferential effusion; moderate, 15–19mm of loculated effusion or 10–14mm of circumferential effusion; and large, any amount more than moderate effusion. This grade was determined during the diastolic cardiac phase [10].

Cardiac tamponade was suspected in patients with otherwise unexplained tachycardia, hypotension, or clinical signs including pulsus paradoxus, raised jugular venous pressure, and cardiomegaly. The diagnosis was supported by following findings on echocardiography; right or left atrial and ventricular collapse, inferior vena cava distension, and respiratory flow variation of mitral and tricuspid inflow velocities [10]. To exclude acute pericardial hemorrhage related to bleeding complications immediately after surgery, ten patients (1.8%) who developed cardiac tamponade within 48 hours after surgery or continuous blood loss though pericardial tube were excluded in the analysis.

Urgent interventions were conducted for every patient with cardiac tamponade following the diagnosis. The selection of pericardial drainage method depended on the clinicians’ decision. In general, surgical drainage was preferred in patients with loculated PE or PE with posterior-dominant location. Otherwise, percutaneous catheter drainage under echocardiographic or fluoroscopic guidance was conducted.

Overall, the median follow-up duration was 34.8 months. The median time of pericardial catheter drainage or surgical drainage following valvular surgery was evaluated. Readmission or emergency department visit was counted only when requiring medical care from the department of cardiology or cardiovascular surgery after discharge. Recurrence of PE was defined when the development of a small or greater amount of effusion was identified on echocardiography following drainage of PE during the follow-up period.

Continuous variables were presented as a mean ± standard deviation and categorical variables as absolute and relative frequencies (%). The differences between the two groups with or without cardiac tamponade were analyzed using the Student t-test for continuous variables and the Chi-square statistic for categorical variables. Univariate and multivariate Cox model analyses were used to investigate the relationship between clinical, valvular, operative, or echocardiographic characteristics, and the occurrence of cardiac tamponade. Univariate Cox regression analysis was first performed with each variable and statistically significant variables were selected (p<0.010). For multivariate Cox regression analysis, model 1 included infective endocarditis, mechanical valve replacement, and concomitant MAZE procedure as covariates. Then, any amount of PE on the first postoperative TTE was added in the final Cox regression model (model 2). Cardiac tamponade-free survival rates were generated using Kaplan-Meier curves and compared with the log-rank test according to the presence of infective endocarditis, mechanical valves, concomitant MAZE procedure, or any amount of PE at the first postoperative TTE. For all tests, statistical significance was set at p<0.05. All statistical analyses were performed with SPSS version 21.0 (SPSS Inc., Chicago, Illinois).

## Results

The baseline characteristics of all 556 patients and the two groups according to the occurrence of cardiac tamponade are shown in Table 1. The mean age was 58.5 years, and 48.7% were male. The numbers of patients with rheumatic valve disease, degenerative valve disease and infective endocarditis as the etiology of valvular dysfunction were 167 (30.0%), 333 (59.9%), and 44 (7.9%), respectively. Bicuspid aortic valve was surgically identified in 86 patients (15.5%). Two hundred sixty-six (47.9%) patients had undergone valve replacement with a mechanical prosthesis. Concomitant aorta surgery, CABG, and MAZE surgery were performed in 58 (10.4%), 56 (10.1%) and 14 (2.5%) patients, respectively. There were no significant differences in demographic characteristics, proportion of underlying valve function, or concomitant surgery between the two groups based on the occurrence of cardiac tamponade. However, the prevalence of infective endocarditis as a reason for heart valve surgery was significantly higher in patients with cardiac tamponade than those without (20.8% *vs*. 7.3%, *P* = 0.017). In terms of types of surgery, more patients in the cardiac tamponade group had received mechanical valve replacement compared with the other group (66.7% *vs*. 46.1%, *P* = 0.044).

**Table 1:** Characteristics of study population.

	All (n = 556)	Cardiac Tamponade	
		No (n = 532)	Yes (n = 24)
**Demographic characteristics**			
Age, years ± SD	58.5 ± 12.9	58.7 ± 12.9	54.3 ± 12.8
Male gender, n (%)	271 (48.7)	256 (48.1)	15 (62.5)
Body mass index, kg/m^2^ ± SD	23.2 ± 3.1	23.2 ± 3.2	23.2 ± 2.4
Prior history of valve operation, n (%)	41 (7.4)	41 (7.7)	0 (0)
**Etiology of valvular disease**			
Rheumatic valve disease, n (%)	167 (30.0)	160 (30.1)	7 (29.2)
Degenerative valve disease, n (%)	225 (40.5)	219 (41.2)	6 (25.0)
Secondary valve disease, n (%)	13 (2.3)	13 (2.4)	0 (0.0)
Prosthetic valve failure, n (%)	21 (3.8)	21 (3.9)	0 (0.0)
Infective endocarditis, n (%)	44 (7.9)	39 (7.3)	5 (20.8)*
Bicuspid aortic valve, n (%)	86 (15.5)	80 (15.0)	6 (20.8)
**Underlying valve function**			
Mitral regurgitation, n (%)	133 (23.9)	129 (24.2)	4 (16.7)
Mitral stenosis, n (%)	167 (30)	160 (30.1)	7 (29.2)
Aortic regurgitation, n (%)	67 (12.1)	64 (12)	3 (12.5)
Aortic stenosis, n (%)	130 (23.4)	125 (23.5)	5 (20.8)
Tricuspid regurgitation, n (%)	3 (0.5)	3 (0.6)	0 (0.0)
**Types of surgery**			
Replacement with mechanical prosthesis	261 (46.9)	245 (46.1)	16 (66.7)*
Aortic valve, n (%)	157 (28.2)	146 (27.4)	11 (45.8)
Mitral valve, n (%)	146 (26.3)	137 (25.8)	9 (37.5)
Double valves, n (%)	49 (8.8)	45 (8.5)	4 (16.7)
Replacement with bioprosthesis	159 (28.6)	155 (29.1)	4 (16.7)
Aortic valve, n (%)	123 (22.1)	120 (22.6)	3 (12.5)
Mitral valve, n (%)	52 (9.4)	51 (9.6)	1 (4.2)
Double valves, n (%)	16 (2.9)	16 (3.0)	0 (0.0)
Repair			
Aortic valve, n (%)	3 (0.5)	3 (0.6)	0 (0.0)
Mitral valve, n (%)	146 (26.3)	141 (26.5)	5 (20.8)
Concomitant surgery			
Aorta surgery, n (%)	58 (10.4)	55 (10.3)	3 (12.5)
CABG, n (%)	56 (10.1)	55 (10.3)	1 (4.2)
MAZE operation, n (%)	14 (2.5)	12 (2.3)	2 (8.3)

* for *p* value < 0.05

CABG, coronary artery bypass grafting

**Fig 1:**
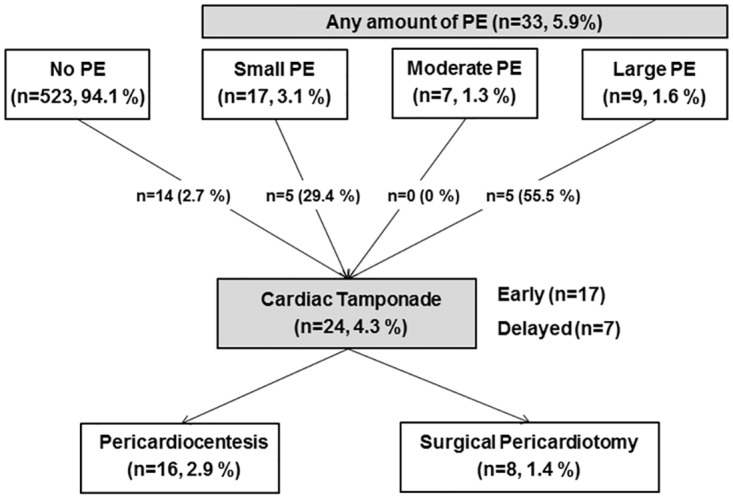
Development and treatment of cardiac tamponade according to post-operative 5-day pericardial effusion: Pericardial effusion was measured median 5-day after valvular surgery. Early cardiac tamponade occurred within 30 days after surgery. Delayed tamponade was defined when it occurred 30days after surgery. Cardiac tamponade did not occur in patients with moderate PE at 5-day trans-thoracic echocardiography.

Thirty-three patients (5.9%) exhibited PE at TTE performed at a median of 5 days following heart valve surgery (Table 2, Fig 1). Twenty-four (4.3%) patients developed cardiac tamponade on serial echocardiography. Among these, 17 patients experienced cardiac tamponade within 30 days following heart valve surgery, and 7 patients after 30 days. Furthermore, 29.4% (5/17) of patients with small PE and 55.5% (5/9) patients with large PE at the first postoperative TTE developed cardiac tamponade that required an urgent procedure. Among the 24 patients who exhibited cardiac tamponade, 16 patients underwent pericardiocentesis and 8 patients received surgical pericardiotomy. The median time of percutaneous or surgical pericardial drainage following heart valve surgery was 17 (IQR 13–30) days. There is no difference between groups with and without cardiac tamponade in postoperative laboratory findings including inflammatory markers, cardiac biomarkers, and coagulation profiles.

**Table 2:** Echocardiographic and laboratory characteristics.

	All (n = 556)	Cardiac Tamponade	
		No (n = 532)	Yes (n = 24)
**Pericardial effusion**			
No (< small), n (%)	523 (94.1)	509 (95.7)	14 (58.4)
Any amount, n (%)	33 (5.9)	23 (4.3)	10 (41.7) *
Small, n (%)	17 (3.1)	12 (2.3)	5 (20.8)*
Moderate, n (%)	7 (1.2)	7 (1.3)	0 (0.0)
Large, n (%)	9 (1.6)	4 (0.7)	5 (20.8)*
**Timing of cardiac tamponade**			
Early (< 30 days), n (%)	17 (3.1)	0 (0)	17 (70.8)
Delayed (≥ 30days), n (%)	7 (1.3)	0 (0)	7 (29.2)
**Postoperative laboratory findings**			
WBC count, 10^9^/mL	20.1 ± 7.3	20.2 ± 7.3	19.1 ± 7.2
CK-MB, IU/L	26.6 ± 22	26.2 ± 21.7	33.4 ± 26.9
CK, IU/L	621.5 ± 498.4	617.7 ± 499.6	692.9 ± 480.4
CRP, mg/L	90.8 ± 34	91.5 ± 33.9	78.5 ± 34.1
aPTT, sec	45.5 ± 15.7	45.5 ± 15.9	43.4 ± 13.6
PT, INR	1.83 ± 0.74	1.83 ± 0.75	1.71 ± 0.63
Number of patients receiving supra-therapeutic range of anticoagulation^#^, n (%)	23 (4.1)	23 (4.3)	0 (0.0)

* for *P* value < 0.05

^#^Supratherapeutic range of anticoagulation was defined as follows: aPTT > 80 seconds at post-operative 3day or PT > 4 INR at the day of the 1^st^ post-operative echocardiography.

WBC, white blood cell; CK, creatine kinase; CRP, C-reactive protein; aPTT, activated partial thromboplastin time; PT, prothrombin time; INR, international normalized ratio

Table 3 shows the results from the Cox regression analyses. In univariate analysis, infective endocarditis as the etiology of valve disease, mechanical valve replacement, and any amount of PE at the first postoperative TTE were related to the occurrence of cardiac tamponade. A concomitant MAZE procedure showed marginal significance in univariate analysis. In multivariate Cox regression model 1, not including postoperative TTE findings, the occurrence of cardiac tamponade was associated with infective endocarditis (hazard ratio, HR, 3.21; confidence interval, CI, 1.17–8.78, *P* = 0.023) and a concomitant MAZE procedure (HR 4.55; CI 1.05–19.71, *P* = 0.043). After including any amount of PE at the first postoperative TTE in model 2, mechanical valve replacement (HR 2.69; CI 1.13–6.38, *P* = 0.025) and any amount of PE (HR 14.00; CI 5.90–33.18, *P*<0.001) were independently related to the occurrence of cardiac tamponade.

**Table 3:** Factors associated with postoperative cardiac tamponade.

Variable	Univariate analysis		Multivariate analysis			
			Model 1		Model 2	
	HR (95% CI)	*P* value	HR (95% CI)	*P* value	HR (95% CI)	*P* value
**Demographic characteristics**						
Age	0.98 (0.95 to 1.01)	0.111				
Male gender	1.79 (0.78 to 4.01)	0.168				
BMI	0.99 (0.87 to 1.13)	0.894				
**Valve characteristics**						
Infective endocarditis	3.38 (1.26 to 9.07)	0.015	3.21 (1.17 to 8.78)	0.023	2.26 (0.82 to 6.26)	0.116
Bicuspid aortic valve	1.80 (0.72 to 4.54)	0.212				
**Type of operation**						
Mechanical valve replacement	2.35 (1.01 to 5.50)	0.048	2.15 (0.91 to 5.07)	0.080	2.69 (1.13 to 6.38)	0.025
Concomitant MAZE	3.79 (0.89 to 16.13)	0.071	4.55 (1.05 to 19.71)	0.043	1.33 (0.29 to 6.20)	0.717
**Postoperative PE**						
Any amount (≥ small)	13.96 (6.20 to 31.47)	< 0.001	-	**-**	14.00 (5.90 to 33.18)	<0.001

**Fig 2:**
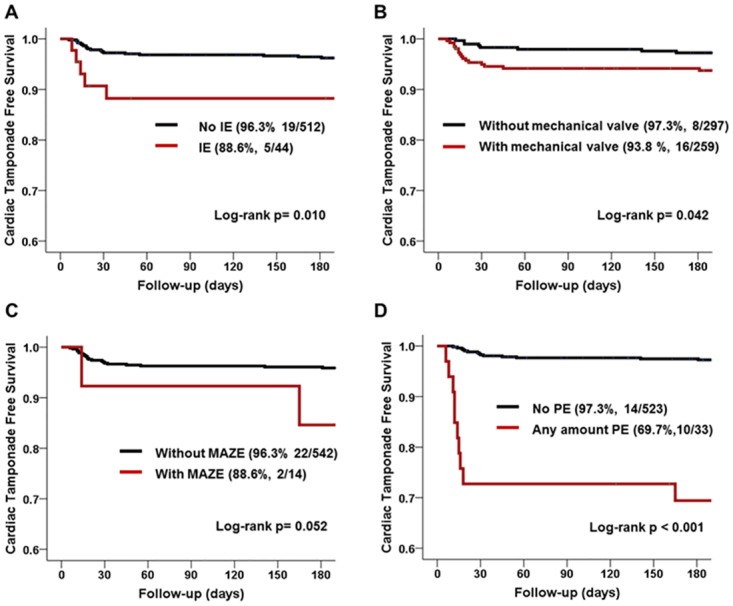
Kaplan-Meier curve for cardiac tamponade-free survival between groups with and without risk factors (A: infective endocarditis, B: mechanical valve replacement, C: MAZE operation, D: Any amount of pericardial effusion on the first postoperative echocardiography): Infective endocarditis, mechanical valve replacement, and any amount of PE at the first postoperative TTE were associated with decreased cardiac tamponade-free survival. Concomitant MAZE procedure showed marginal statistical significance (*P* = 0.052) in cardiac tamponade-free survival, and delayed cardiac tamponade also occurred even after 30 days after surgery.

Kaplan-Meier curves for cardiac tamponade-free survival are depicted in Fig 2. Infective endocarditis (*P* = 0.010), mechanical valve replacement (*P* = 0.042), and any amount of PE at the first postoperative TTE (*P*<0.001) were associated with decreased cardiac tamponade-free survival, and the cardiac tamponade mostly occurred within 30 days after heart valve surgery. Concomitant MAZE procedure showed marginal statistical significance (*P* = 0.052) in cardiac tamponade-free survival, and delayed cardiac tamponade also occurred even after 30 days after surgery. The number of mean hospital days was higher in patients with cardiac tamponade than in those without (34.9 *vs*. 13.5, *P* = 0.031). After pericardial drainage, there was no echocardiographic recurrence of PE more than small amount during a median of 34.8 (IQR 14.9–43.7) months after surgery.

## Discussion

The principal findings in the present study are that 1) the overall incidence of cardiac tamponade was 4.3% after heart valve surgery; 2) the occurrence of cardiac tamponade was related with infective endocarditis, valve replacement using mechanical valve, concomitant MAZE procedure, and any amount of PE at the first postoperative TTE, which was performed about 5 days after surgery; 3) mechanical valve replacement and any amount of PE at the first postoperative TTE were independent predictors for the occurrence of cardiac tamponade; 4) cardiac tamponade shows a benign clinical course without recurrence after timely intervention. The results of our study indicate that patients with any amount of PE at the first postoperative TTE or mechanical valve replacement should get more attention in the occurrence of cardiac tamponade. In other words, patients without a mechanical valve and pericardial effusion at the first postoperative TTE were less likely to experience cardiac tamponade in the postoperative period.

The incidence of cardiac tamponade (4.3%) in this study was higher than those observed in previous studies, which reported that only 0.8–0.9% patients experienced cardiac tamponade after open heart surgery [2,10]. The present study included only patients undergoing heart valve surgery, which is associated with increased risk of PE and consequent cardiac tamponade compared to CABG alone [2,3,10].

Although a few potential mechanisms have been suggested in previous studies, the exact pathophysiology that results in postoperative PE or occurrence of cardiac tamponade is still unclear. The results of this study yield some insight into the pathophysiology of postoperative PE and consequent cardiac tamponade following heart valve surgery.

First, Anticoagulation after heart valve surgery did not have obvious association with increased risk of cardiac tamponade in this study, which has been considered a risk for cardiac tamponade by promoting pericardial bleeding [2,10]. In this study, the levels of postoperative anticoagulation including both aPTT and international normalized ratio (INR) in patients with cardiac tamponade were not different from these values in patients without cardiac tamponade. The exclusion of immediate cardiac tamponade occurring within 48 hours after surgery, which is usually associated with pericardial bleeding, may influence the lack of the association between anticoagulation and the occurrence of cardiac tamponade. Moreover, since every patient received oral anticoagulants at least for 3 to 6 months after heart valve surgery if there was no absolute contraindication, it was a challenge to prove the impact of anticoagulation on the occurrence of cardiac tamponade. However, the relationship between mechanical valve replacement and cardiac tamponade might indicate an effect of anticoagulation levels on the occurrence of cardiac tamponade.

Second, postoperative PE has been regarded as one of the major components of PPS, which is characterized by lasting fever, pleuritic symptoms, and pericardial or pleural effusions following open heart surgery. Imazio *et al*. reported that PE was observed in about 90% of patients with PPS [1]. Various studies have suggested that immunopathic mechanisms are the most likely mechanism leading to the development of PPS [11,12]. Therefore, it is possible that increased both local and systemic inflammation following surgery may play a role in the development of PE and cardiac tamponade. Mechanical valves can promote both systemic and local inflammation, represented by serum inflammatory markers such as IL-6 [13], and metallosis of the adjacent tissue [14]. Infective endocarditis is undoubtedly proinflammatory in status [15]. Previous studies demonstrated higher incidence, associated factors, and poor clinical outcomes of PE in patients with infective endocarditis [16,17]. High titers of circulating immune complexes in patients with infective endocarditis may aggravate PPS and result in cardiac tamponade. A concomitant MAZE procedure also enhances local inflammation caused by either surgical incision or the ablation itself [18]. Patients undergoing surgical or catheter ablation for atrial fibrillation exhibited an inflammatory response after the procedure [19,20]. Furthermore, a recent worldwide survey on the efficacy and safety of catheter ablation for atrial fibrillation reported the occurrence of cardiac tamponade a median of 12 days following the ablation procedure [21]. Thus, infective endocarditis and a concomitant MAZE procedure may raise pericardial and systemic inflammation, which could mediate the relationship between cardiac surgery and the development or worsening of PE.

The important role of postoperative TTE in screening the risk of cardiac tamponade after heart valve surgery should be highlighted. In this study, the most powerful predictor for the occurrence of cardiac tamponade was any amount of PE at the first postoperative TTE. Therefore, postoperative TTE after heart valve surgery is very useful for the identification of patients who are at risk of occurrence of cardiac tamponade and tailored frequent clinical or echocardiographic follow-up will be helpful for the early detection of, and timely intervention against, cardiac tamponade. In particular, delayed cardiac tamponade may develop silently in the absence of obvious clinical signs. Because it can be easily missed, and without early diagnosis and treatment, can be life threatening, early decompression is required as soon as its presence is confirmed [22,23]. Among the study population, there was no fatal event due to cardiac tamponade following timely intervention.

Postoperative cardiac tamponade prolonged the patient’s hospital stay and was related to a high rate of cardiovascular readmission. Postoperative constriction is associated with PE [6,7], which can be associated with high readmission rate in patients with PE. Generally, however, cardiac tamponade has a benign clinical course without recurrence following timely intervention.

To date, there is no obvious way to prevent cardiac tamponade following open heart surgery. Erdil’s group suggested that posterior cardiotomy during valve replacement operation might reduce the risk of cardiac tamponade, but they failed to show statistical significance in their study [24]. Imazio *et al*. demonstrated that prophylactic colchicine could prevent incident PE and the worsening of PE as well as PPS [25]. It might be particularly helpful to use prophylactic colchicine for preventing cardiac tamponade in high-risk patients, such as those with infective endocarditis or those with planned surgery involving a mechanical prosthesis or concomitant MAZE procedure. A prospective study is warranted to validate the benefit of preventive medical treatment in patients at high risk of cardiac tamponade following heart valve surgery.

Several limitations should be addressed in this study. First, regular echocardiographic follow-up was not strictly followed, because of the retrospective nature of the study. It is possible that some cases with PE were missed. Second, the incidence of cardiac tamponade in this study was calculated after the exclusion of ten patients who had suspicious immediate post-operative pericardial hemorrhage. The incidence of cardiac tamponade including immediate bleeding seems to be a bit underestimated. Treatment of PE that was lower than the amount that causes cardiac tamponade was different in each patient, and ranged from observation to cessation of anticoagulation or adding anti-inflammatory medication. Finally, only C-reactive protein was measured to represent systemic inflammatory reaction, which could be suboptimal to demonstrate systemic inflammation.

## Conclusions

Cardiac tamponade is more common in patients with any amount of PE on the first postoperative echocardiography after valvular heart surgery or mechanical valve replacement. Although postoperative cardiac tamponade prolongs hospital stay and requires more readmission compared with those without, it has a benign clinical course without recurrence following timely intervention.

## Abbreviations

PE: pericardial effusion
PPS: post-pericardiotomy syndrome
CABG: coronary artery bypass grafting
WBC: white blood cell count
CK: creatine kinase
CRP: C-reactive protein
aPTT: activated partial thromboplastin time
PT: prothrombine time
TTE: trans-thoracic echocardiography
